# Rural and Non-Rural Digital Divide Persists in Older Adults: Internet Access, Usage, and Perception

**DOI:** 10.1093/geroni/igaa057.1329

**Published:** 2020-12-16

**Authors:** Hee Yun Lee, Eun Young Choi, Youngsun Kim, Jessica Neese, Yan Luo

**Affiliations:** 1 University of Alabama, Tuscaloosa, Alabama, United States; 2 Leonard Davis School of Gerontology, Los Angeles, California, United States; 3 Kyunghee University, Yongin-si, Republic of Korea; 4 MSW, Tuscaloosa, Alabama, United States

## Abstract

Despite the overall increase in Internet use among older adults, the digital divide within older Americans remains substantial. This trend is particularly true for older adults living in rural areas. Informed by the Social Determinants of Health Framework, our study aims to examine how one’s residential area relates to (1) Internet Access, (2) subtypes of usage patterns, and (3) perceptions on technology use. Cross-sectional data were drawn from the 2012 Health and Retirement Study (HRS). The sample consisted of 18,196 older adults aged 50 and above (47.6% rural residents). A series of linear and logistic regression analyses were performed. Our models controlled for demographic characteristics, socioeconomic status, and health conditions. Compared to older adults living in urban areas, those residing in rural areas had 29% lower odds of internet access. Living in rural areas predicted lower levels of all sub-types of technology use (communication, financial, health, and media technology). In addition, non-users in rural areas showed more unfavorable perceptions of technology than urban residents. They were more likely to conceive technology as “too complicated”, “too hard to learn”, and “too difficult to keep up with all changes.” Our findings suggest that substantial segments of older adults in rural areas are still behind in accessing and adopting digital technology. Targeted intervention efforts are urgently needed to reduce technology inequality including comprehensive plans to expand broadband access and building mobile technology infrastructure for rural communities.

